# Exploring the Potential of Human Milk and Formula Milk on Infants’ Gut and Health

**DOI:** 10.3390/nu14173554

**Published:** 2022-08-29

**Authors:** Hui-Yuan Chong, Loh Teng-Hern Tan, Jodi Woan-Fei Law, Kar-Wai Hong, Vanassa Ratnasingam, Nurul-Syakima Ab Mutalib, Learn-Han Lee, Vengadesh Letchumanan

**Affiliations:** 1 Novel Bacteria and Drug Discovery Research Group (NBDD), Microbiome and Bioresource Research Strength (MBRS), Jeffrey Cheah School of Medicine and Health Sciences, Monash University Malaysia, Bandar Sunway 47500, Malaysia; 2 Clinical School Johor Bahru, Jeffrey Cheah School of Medicine and Health Sciences, Monash University Malaysia, Johor Bahru 80100, Malaysia; 3 UKM Medical Molecular Biology Institute (UMBI), Universiti Kebangsaan Malaysia, Kuala Lumpur 56000, Malaysia; 4 Faculty of Health Sciences, Universiti Kebangsaan Malaysia, Kuala Lumpur 56000, Malaysia

**Keywords:** gut microbiota, infant, human milk, formula milk, immune system

## Abstract

Early-life gut microbiota plays a role in determining the health and risk of developing diseases in later life. Various perinatal factors have been shown to contribute to the development and establishment of infant gut microbiota. One of the important factors influencing the infant gut microbial colonization and composition is the mode of infant feeding. While infant formula milk has been designed to resemble human milk as much as possible, the gut microbiome of infants who receive formula milk differs from that of infants who are fed human milk. A diverse microbial population in human milk and the microbes seed the infant gut microbiome. Human milk contains nutritional components that promote infant growth and bioactive components, such as human milk oligosaccharides, lactoferrin, and immunoglobulins, which contribute to immunological development. In an attempt to encourage the formation of a healthy gut microbiome comparable to that of a breastfed infant, manufacturers often supplement infant formula with prebiotics or probiotics, which are known to have a bifidogenic effect and can modulate the immune system. This review aims to elucidate the roles of human milk and formula milk on infants’ gut and health.

## 1. Introduction

The gut microbiome composition in neonates is closely connected with events such as how they are born (full-term or preterm), the mode of delivery (vagina delivery or caesarean section), what neonates are fed (human or formula milk), and how neonates are cared for (mother’s care or at neonatal intensive care unit (NICU)) [1]. The early microbiome colonization in the infants’ gut plays a vital role in shaping and maintaining future health outcomes [1,2,3]. Studies have suggested that early-life microbiota can predict the risk of developing illnesses such as atopic diseases, obesity, and type 1 diabetes [4,5,6]. It was previously believed that the onset of microbial colonization of the baby’s gut is at birth. However, recent evidence indicates the presence of microbial communities in the placenta, amniotic fluid, umbilical cord, and meconium, challenging the traditional view of the sterile in utero environment [7,8,9,10]. While still controversial, these findings suggest that the infant’s onset of microbial transfer and gut colonization process may begin prenatally [11,12].

The development of infants’ gut microbiota begins at birth and continues to be shaped up until two–three years, reaching a relatively stable and typical adult microbial taxonomic makeup [13]. The neonates are first exposed to the maternal microbiome community. The maternal microbiome reservoir colonizes the gut through vertical transmission and microbial taxa obtained from the external surroundings [14,15]. After birth, the neonatal gut microbiome is briefly dominated by *Staphylococcaceae* or *Enterobacteriaceae* before *Bifidobacteria* become predominant [16]. *Bifidobacterium* has long been known to confer health benefits to the host. A higher level of *Bifidobacterium* in the infant’s gut is associated with a lower risk of childhood infections, atopic disorders, and obesity [17,18]. After weaning, the infant gut microbiome transitions from a *Bifidobacteriaceae*-dominated microbiota to an adult-like composition. The key factor influencing these changes in microbiota composition is breastfeeding cessation rather than the introduction of complementary feeding [19,20]. By the age of three, a stable adult-type gut microbiota, which is clustered into three enterotypes—*Bacteroides*, *Prevotella*, *Ruminococcus*—is acquired [21,22].

Soon after birth, the infants are fed with human milk or formula milk. It is widely agreed that human milk is the best food for babies, with health effects driven by the combined nutritional and bioactive components. The short-term and long-term health benefits from breastfeeding based on the nutritional, physiological, and development perspectives are well established. The World Health Organization (WHO) and the United Nations Children’s Fund (UNICEF) strongly recommend breastfeeding babies within the first hour of birth and continuing breastfeeding for the first six months, without any other solid food or water [23,24]. Likewise, the American Academy of Pediatrics (AAP) also suggests breastfeeding for 12 months or beyond while the infant is started on complementary food [25]. Human milk comprises beneficial bacterial species that contribute to establishing the baby’s gut microbiome and play a part in infection prevention and immunomodulation [26]. Besides containing all the essential nutrients to fulfil the nutritional requirements for optimal growth of the infant, human milk also contains bioactive components—oligosaccharides, immunoglobulins, hormones, growth factors, cytokines and chemokines—that play important roles in the microbiome and immune system development, as well as maintaining the gut mucosal barrier function [27]. Given their implications for health and immune development and their potential for therapeutic manipulation, the human milk microbiome and bioactive compounds have become an area of interest for research [28]. Although human milk is the best for babies, alternatives are sought when human milk is insufficient, babies who cannot be fed with human milk, or should not receive milk from their mothers (due to health reasons) [29]. In these circumstances, babies are fed with formula milk that mimics the composition of human milk in terms of micronutrients and macronutrient content. Hence, this narrative review aims to discuss the role of human milk and formula milk on infants’ gut and health.

## 2. Factors Influencing Infants’ Gut

### 2.1. Mode of Delivery

Many perinatal variables have been shown to contribute to infant gut microbiota development, such as mode of delivery, gestational age, infant diet, use of antibiotics, and infant hospitalization. The initial microbes that colonize the infants’ gut come from their mother and, if vaginally born, then the microbes are from the vaginal microbiome of the mother. Dominguez-Bello and colleagues studied bacteria sampled from infants right after birth and compared them with samples from different maternal body sites. They revealed that all the babies shared similar microbiome composition. If vaginally born, then the bacteria source is from the mother’s vagina [30]. However, Ferreti et al. reported that neonates’ oral and gut microbiomes at one–three days postpartum do not resemble the microbiome taxa from a specific mother’s body site. Some neonates had taxonomic makeup that resembled the vaginal microbiome, while some had the same composition as the mothers’ faecal microbiome. The early colonization is suggested to be influenced by stochastic events [31]. Infants delivered by vaginal or caesarean are usually exposed to different microbiome communities. In the first three weeks of life, vaginally born babies are exposed to their own mothers’ vaginal microbiomes and are enriched with *Bifidobacterium*, *Escherichia*, *Lactobacillus* and *Bacteroides* species. Whereas caesarean-delivered babies are exposed to microbiome species of the skin of parents and potentially colonized by species from hospital environments. Caesarean-born babies have a gut that is commonly colonized by *Enterobacter*, *S. epidermidis*, *K. pneumoniae*, *E. coli* and *Klebsiella* [32,33,34].

The effect of birth mode on gut microbial colonization and diversity is most significant during the first six months of life, albeit decreasing with age [33,35]. Wampach et al. studied the microbial taxa in 33 mother–infant pairs (MIPs) at an interval of the first, third, and fifth days postpartum. They observed the vaginally born infants had a higher number of vertically transmitted strains compared to those born via caesarean. The MIPs shared 23 taxa mostly *Bacteroides* and *Bifidobacterium* in vaginally delivered infants [36]. Similarly, another study by Makino and colleagues found that the majority of vaginally born babies share a *Bifidobacterium* strain profile with the maternal gut microbiome. The caesarean-born babies did not exhibit any strain sharing profiles with the maternal gut [37]. A study also found that at the early stage of vaginal delivery, the infant’s gut is enriched with *Lactobacillus*, and then the *Bacteroides* will increase in the second week. These microbial patterns are not observed in caesarean-born infants [30]. Based on these studies, delivery mode does play a significant role in enriching the gut microbiota of infants.

### 2.2. Gestational Age and Administration of Antibiotics

The gestational age at birth is another crucial factor in the development of neonates’ gut microbiome. The intestines of preterm neonates born around 22 to 36 weeks of gestation are more permeable than term neonates (37 to 42 weeks of gestation). A lower abundance of *Bifidobacterium* and *Bacteroidetes* and a higher abundance of *Enterobacteriaceae*, *Enterococcaceae*, and *Lactobacillaceae* were observed in preterm neonates compared to term neonates [38]. Korpela et al. studied the faecal samples from 45 preterm neonates to explore the gut microbiota properties and development in preterm neonates. The study found that preterm neonates’ gut exhibited a lower number of predominant bacteria genera such as *Bifidobacterium*, *Enterobacter*, *Staphylococcus*, or *Enterococcus*. They suggested that breastfeeding may help preterm infants in the hospital acquire a normal gut microbiota similar to that of infants who are born to term [39].

Perinatal antibiotic usage is also known to affect the gut microbial composition, as indicated by a reduced gut microbiome diversity and stability [40,41]. A study comparing the gut microbial colonization in infants exposed to perinatal antibiotics and infants who had no antibiotics exposure reported the differences in gut microbiome between the exposure groups and control group, which persist at six months of age and are not prevented by consumption of probiotic *Lactobacillus reuteri* [42]. Studies have shown a reduced *Bifidobacterium* abundance in term babies upon antibiotic treatment for Group B *Streptococcus*. The other bacteria community (*Lactobacillus* sp., *Bacteroides fragilis*, and *Clostridium difficile*) were not affected [43,44].

### 2.3. Feeding Mode

Another essential factor influencing the infant’s gut microbial colonization and composition is the mode of infant feeding. While infant formula milk has been designed to resemble breast milk as much as possible, the gut microbiome composition in breastfed and formula-fed infants remains distinct. Several studies have demonstrated the beneficial effects of human milk on the disrupted microbiome caused by other aforementioned perinatal factors. For example, limited restoration of the disturbed gut microbiota was demonstrated by Liu et al. in infants born through caesarean section by exclusive breastfeeding compared to partial breastfeeding [45]. Cong et al. studied the influence of feeding on the early life gut microbial composition of preterm babies and found that human milk enriched microbial diversity and helped establish a balanced microbial community structure [46]. In breastfed term infants, a higher abundance of *Bifidobacterium* was observed, whereas formula milk fed infants had an increase in *Enterobacteriaceae*, *Bacteroidaceae*, and *Clostridiaceae* [19,47].

## 3. Human Milk

### 3.1. Human Milk Composition

Human milk contains both nutritive and non-nutritive components, which promote normal growth and contribute to immunological development. The nutritional components are classified into macronutrients (carbohydrate, protein, and fat) and micronutrients (minerals and vitamins) [48]. Human milk contains approximately 7.0% lactose, which is a disaccharide. As the predominant carbohydrate in human milk, lactose contributes to 40% of the milk’s gross energy [49]. In smaller amounts, other carbohydrates in milk are monosaccharides, such as glucose and galactose; disaccharides, such as lactulose; oligosaccharides and some polysaccharides [49]. While oligosaccharides in human milk are non-nutritive and indigestible, they play an important role in infant gut microbiome development [50,51]. The protein content in human milk is estimated to be 0.9%–1.2% [49,52]. Approximately 30% of the total protein is casein, and 70% is whey proteins primarily constituted by lactoferrin, alpha-lactalbumin, and secretory IgA [49]. The mean fat concentration in mature milk is approximately 3.8%, contributing to about half of the total energy provided by human milk (Table 1) [49,53].

The composition of breast milk is dynamic and changes in response to the baby’s needs. A study by Paulaviciene et al. showed that human milk exhibits marked circadian fluctuations in the composition of macronutrients, particularly protein and fat, and the diurnal variations are more evident in the breast milk of mothers of premature babies [56]. The human milk nutritional composition also changes within each feed; for example, there are more fat and energy contents in hindmilk compared to foremilk [57,58]. Human milk has three stages, and its composition varies across the different lactation stages. The first milk secreted immediately after delivery of a newborn is known as colostrum, which differs from the milk produced later by containing higher concentrations of proteins and immunological components such as lactoferrin, leukocytes, immunoglobulins, and growth factors, as well as containing lower concentrations of carbohydrates and fats [59,60,61,62]. The second stage lasts from day 6 to day 14 postpartum, and the milk produced is known as transitional milk [63]. There is an increase in milk production, carbohydrate, and lipid content in this stage, such that the infant’s nutritional requirements can be met for rapid growth and development [27,64]. The milk is considered mature by day 15 to day 30 postpartum, and its composition fluctuations are relatively less [63]. Mature milk is richer in lactose and fat, but its protein is at a lower concentration than colostrum [62]. Some studies have demonstrated higher carbohydrate, protein, fat, and energy contents in preterm milk compared to term milk. However, some authors found higher protein levels in preterm milk, whereas other macronutrient contents were not significantly influenced by gestational age [57,61,65,66]. Two recent studies have reported that the macronutrient composition is not affected by the degree of prematurity [67,68]. Feeding practices may also be predictors of the nutrient composition as indicated by lower fat concentration, lower total calorie content, and higher carbohydrate and protein contents in the milk of mixed-feeding mothers compared to those who exclusively breastfed [69].

#### 3.1.1. Oligosaccharides

Human milk oligosaccharides (HMOs), the third most abundant human milk component, are present at approximately 0.8–1.4% [49]. The concentration of HMOs may vary with the stage of lactation, as reflected by a higher level of most HMOs in colostrum, and reduces as lactation progresses [70,71,72,73]. A higher level of HMOs is also found in the milk of mothers of preterm babies than in mothers of term babies [74]. While they are indigestible by infants, HMOs have prebiotic effects and are metabolized by certain gut bacteria, promoting their growth and colonization within the infant’s gut [75]. De Leoz et al. demonstrated a shift in the infant gut microbiota from non-HMO-utilizing bacteria to HMO-utilizing bacteria after receiving breast milk for a few weeks. There was also a reduction in faecal HMOs as the abundance of HMO-consumers increased, confirming the utilization of HMOs by the gut bacteria [76]. *Bifidobacteria* and *Bacteroides* strains have a high capacity in metabolizing HMOs and utilizing them as a source of energy. In contrast, other species such as *Enterococcus*, *Clostridium*, *Escherichia coli*, *Staphylococcus*, and *Streptococcus* are inefficient in HMOs metabolism [77,78]. By selectively encouraging the growth of beneficial bacteria over potential pathogens, HMOs may help to prevent infections.

Furthermore, oligosaccharides fermentation by gut bacteria leads to the production of short-chain fatty acids (SCFAs) such as acetate and organic acids such as lactate, generating a lower pH environment that restrains the growth of enteric pathogens and promotes the absorption of nutrients [79,80]. In addition, *Bifidobacterium* grown on HMOs is associated with increased anti-inflammatory cytokine and decreased inflammatory gene expression, indicating that HMO growth possesses anti-inflammatory properties [81,82]. The ability of bacteria to adhere to the intestinal epithelium determines the microbial colonization of the infant’s gut. HMOs have been shown to modulate and enhance the ability of *Bifidobacterium infantis* to adhere to intestinal epithelium, which may enhance the bacteria’s colonization ability [81,82,83]. In contrast, HMOs exert anti-adhesive properties against potential pathogens. Studies have demonstrated that some HMOs reduce the level of adhesion of *Campylobacter jejuni*, *Clostridium butyricum*, *Escherichia coli*, *Pseudomonas aeruginosa*, and Norovirus to epithelial cells [84,85,86,87].

#### 3.1.2. Lactoferrin

Lactoferrin, an iron-binding protein, is one of the essential bioactive factors present in human milk [88]. Its concentration changes throughout lactation, with the highest level, found in colostrum and reduces in the later milk until a relatively constant level is reached in mature milk [89,90,91]. Mastromarino et al. established that human milk is the primary source of lactoferrin in the infant’s gut as the level of faecal lactoferrin in breastfed infants was significantly associated with the level of lactoferrin in human milk [92]. Woodman et al. have shown that lactoferrin levels are higher in human milk than formula milk and human lactoferrin showed greater effectiveness in preventing growth of pathogens [93]. Lactoferrin has been shown to exhibit antimicrobial effects on both Gram-positive and Gram-negative bacteria [94]. Tian et al. demonstrated the ability of lactoferrin to inhibit the growth of pathogenic bacteria, such as *Staphylococcus aureus*, *Listeria monocytogenes*, *Salmonella enterica*, and *Escherichia coli.* In contrast, the development of probiotic bacteria such as *Lactobacillus* was not affected [95].

Several mechanisms of the antimicrobial activity of lactoferrin have been described. Mechanisms that have long been known include its iron-binding ability that results in sequestrating and depriving bacteria of iron required for their growth, as well as its ability to interact with lipopolysaccharide of Gram-negative bacteria thus impeding their growth [96,97]. More recent studies have shown that lactoferrin can eliminate biofilms formed by potential pathogens, preventing interactions between microbes and the gut epithelium [98,99]. These characteristics of lactoferrin aid in the enrichment of a healthy gut microbiota in infants.

#### 3.1.3. Immunoglobulins

Immunoglobulins (Ig) are bioactive factors in human milk that offer passive immunological protection to neonates. IgG antibodies cross the human placenta and hence can provide passive immunity to the foetus in utero and the infant postnatally. The IgA and IgM are unable to cross the human placenta; therefore, human milk is an important source of these antibodies, particularly secretory IgA as infants have very low levels of their own IgA at birth and it only gradually rises in the first few months of life when the immune system develops [100]. IgA is the main immunoglobulin in human milk, constituting more than 90% of all milk antibodies, whereas IgM and IgG are present at significantly lower concentrations. The mean concentration of IgG, IgM, and secretory IgA in human milk during the first year was 14.71 mg/L, 3.0 mg/L, and 2.12 g/L, respectively, to Czosnykowska-Łukacka et al. [101]. The levels of IgA and IgM are highest in colostrum and reduce to a relatively stable level in mature milk, whereas the concentration of IgG remains similar throughout the first six months of lactation [102]. A study by Berdi et al. demonstrated a positive correlation between prepregnancy excessive maternal weight and human milk IgM concentration in the first few days of lactation, and smoking during pregnancy is negatively correlated with IgM and IgG2 concentration [103]. Breastfeeding practices may also have an effect on immunoglobulin levels in mother’s milk as evidenced by a higher IgG concentration in the milk of mothers who exclusively breastfed compared to mothers who did not exclusively breastfeed in a study by Abuidhail et al., supporting the recommendation of the World Health Organization to exclusively breastfeed babies for the first six months [104]. IgA and total protein concentrations in human milk have been found to increase after the first year of lactation, indicating that breastfeeding even after introducing food, offers nutritional and immunological benefits to the child. Therefore, prolonged lactation should be encouraged in keeping with the preference of the mother and child [101,105]. IgA helps establish a healthy gut microbiome by enhancing the ability of *Bacteroides* and probiotic strains, such as *Bifidobacterium* and *Lactobacillus*, to adhere to gut epithelium, thereby promoting their colonization [106,107]. Lack of secretory IgA from human milk has been associated with an altered intestinal microbiome and gene expression in the gut epithelium, which may result in greater susceptibility to gut inflammation at a later age [108].

### 3.2. Extracellular Vesicles

Extracellular vesicles (EVs) with their cargos can be detected in any tissue or biofluids, including breast milk. They have been shown to shape the gut microbiome and influence the gut immune response [109]. In vitro study demonstrated intestinal cell uptake of human milk EVs, indicating that EVs are modes of transfer of immunomodulatory genetic material from mother to child [110]. EVs carry a variety of biologically active compounds, such as proteins, lipids, and RNAs [111,112]. Proteomic analysis by van Herwijnen et al. discovered 1963 proteins in EVs from breast milk, and the proteins in EVs were involved in inflammatory signalling pathways [113]. Breast milk was found to have the highest total RNA concentration among other bodily fluids [114]. Human milk contains high levels of immune-related microRNAs, as reported by Kosaka et al. The molecules were found to be stable in very acidic environments, suggesting that miRNAs can endure the gut environment of infants [115]. Zhou et al. also demonstrated that immune-related miRNAs packaged within exosomes in human milk remain impervious to a certain extent when subjected to extreme conditions. It is postulated that these miRNAs are passed to infants from maternal milk through the gastrointestinal tract and are involved in immune system modulation [116]. Alsaweed et al. found a limited number of mature human miRNAs in cow’s milk and soy-based formula, which are expressed at lower levels than those in human milk, indicating that human milk is a richer source of miRNAs for infant immunity and development [117]. EVs in human milk may have therapeutic potential in preventing necrotizing enterocolitis in premature infants. In a study using rat intestinal epithelial cell culture models, there was a significant reduction in the occurrence and severity of experimental necrotizing enterocolitis resulting from administration of EVs derived from breast milk [118]. In addition, Wang et al. and Martin et al. showed that EVs derived from human milk promote gut epithelial cell proliferation and protect intestinal cells from oxidative stress, which may protect infants from necrotizing enterocolitis [119,120].

### 3.3. Human Milk Microbiota

A diverse microbial population is found in human milk, and its microbial composition varies considerably between mothers [121]. A recent study by Kim and Yi detected 392 genera in human milk [122]. Many studies have found *Staphylococcus* and *Streptococcus* to be the predominant genera in human milk [122,123,124,125,126,127]. Pannaraj et al. demonstrated that the divergence in human milk microbiota between mothers increases during the first 24 weeks postpartum and reduces after that when infants are no longer primarily breastfed. The study also showed that in infants who are primarily breastfed (mother’s milk accounts for at least 75% of daily milk intake) during the first month of life, about 40% of the gut microbiota originates from mother’s milk and areolar skin, emphasizing the significance of human milk as a source of bacteria and in determining the infant gut microbiome [128].

Some studies have suggested that factors such as birth mode, gestational age, stages of lactation, and breastfeeding practices may play a role in determining the microbial composition of breast milk. A lower microbiota diversity has been detected in breast milk from women who delivered by caesarean section than those who gave birth by vaginal delivery [123,129]. Gestational age has also been shown to affect the microbial composition of human milk, as evidenced by a much lower abundance of *Bifidobacteria* in preterm breast milk than in term milk [130]. However, a study by Urbaniak et al. found no differences in the human milk microbial profiles attributable to the mode of delivery or gestational age [131]. Regarding the mode of breastfeeding, indirect breastfeeding and pump expression compared to direct breastfeeding and manual expression, respectively, are associated with reduced milk microbiota diversity and richness, higher rates of colonization by potential pathogens, and decreased abundance of *Bifidobacteria* [132]. Gonzalez et al. compared the human milk microbiome in two lactation stages. They found that the predominant species in the early stage were *Streptococcus* and *Staphylococcus*, whereas in the late lactation stage *Pseudomonas* and *Sphingobium* were more abundant [133]. A few studies have shown that microbial richness and diversity in human milk reduce over time from colostrum to mature milk [134,135,136].

## 4. Infant Formula Milk

### 4.1. Types of Infant Formula

Different infant formula products are available, including cow’s milk, soy-based, and specialized formulas such as hypoallergenic and lactose-free. Cow’s milk formula is the most commonly consumed infant formula among other types [137]. Bovine milk contains approximately 3.4% protein, which is significantly higher than human milk, and has a different whey-to-casein ratio of 20:80. Compared to human milk, lactose and lactoferrin levels are lower in bovine milk. Furthermore, human milk has a two-fold higher concentration of alpha-lactalbumin. It does not contain beta-lactoglobulin, whereas beta-lactoglobulin is the dominating protein in the whey fraction of bovine milk [138]. Thirteen oligosaccharides present in human milk were identified in bovine colostrum by Aldredge et al., indicating that bovine milk could be a source of oligosaccharides with bioactivities comparable to those in human milk [139]. However, the concentration and diversity of oligosaccharides in bovine milk are lower than in human milk [140]. Therefore, infant formula manufacturers modify or reformulate cow’s milk to reproduce a nutrient profile similar to human milk [138].

Cow’s milk protein allergy is widely regarded as one of the most common food allergies in the paediatric population [141]. As stated in the food allergy and anaphylaxis guidelines by the European Academy of Allergy and Clinical Immunology, the culprit food allergen should be eliminated from the diet of children with cow’s milk protein allergy. Thus, a therapeutic formula is required. Soy-based formula, extensively hydrolysed formula and amino acid-based formula are alternative infant formulas that can be used in place of cow’s milk formula. While the extensively hydrolysed formula is commonly used as a substitute for cow’s milk, the amino acid-based formula is nonallergenic and may be a substitute when an extensively hydrolysed formula is ineffective [142]. Soy-based formula should only be considered in infants over six months, and if they cannot tolerate extensively hydrolysed formula, if parents cannot afford other specialized formulas, or if the parents strongly prefer it due to veganism [143]. The presence of high concentrations of isoflavones in soy formula has raised concerns about its potential estrogenic effect on infants’ development. Andres et al. evaluated the developmental status of infants receiving different types of milk and revealed that infants fed with soy formula had normal growth and did not differ from cow’s milk formula. However, breastfed infants exhibited better cognitive development than formula-fed infants [144].

### 4.2. Addition of Prebiotics and Probiotics to Infant Formula

To promote a gut microbiome formation comparable to that of a breastfed infant, manufacturers often supplement infant formula with prebiotics and probiotics, which are known to have a bifidogenic effect and can modulate the immune system [145]. Studies have demonstrated that adding prebiotic oligosaccharides to infant formula is well-tolerated in healthy infants and results in softer stools compared to unsupplemented formula [146,147,148,149]. Puccio et al. studied the outcome of supplementing infant formula with two common HMOs—2′fucosyllactose and lacto-N-neotetraose—and reported that the supplemented formula could support proper growth of the infants [150]. Adding these two prebiotic oligosaccharides to infant formula has also been reported to promote the growth of *Bifidobacterium* and produce gut microbial composition closer to breastfed infants [151]. Infant formula supplemented with galacto-oligosaccharides and fructo-oligosaccharides is associated with lower faecal pH and greater abundance of *Bifidobacteria* compared to the unsupplemented formula [152,153]. Castanet et al. revealed that infant formula containing bovine milk-derived oligosaccharides exhibits a bifidogenic effect, helps to modulate the infant gut microbiome, and enhances gut maturation [154].

Probiotics are live microorganisms that provide health benefits to the host when administered in sufficient and appropriate amounts [155]. Supplementing infant formula with probiotic with *Bifidobacterium* sp. and/or lactic acid bacteria such as *Lactobacillus* strains is safe and well-tolerated in healthy term infants [156,157,158]. The supplementation of probiotics increases IgE level in cord blood and promotes TGF-β production. TGF-β is closely related to IgA that is specific to food antigens; thus, elevated TGF-β plays a vital role in atopy prevention in infant early life [159]. The addition of probiotics to infant formula may have beneficial effects on the immunity of infants, as indicated by a decrease in the incidence of the upper respiratory tract and gastrointestinal infections [160]. Infants receiving formula milk supplemented with *Lactobacillus fermentum* had a lower incidence rate of gastrointestinal infections [156,160]. Chi et al. reported that infant formula supplemented with probiotic *Bifidobacterium lactis* promotes enrichment of beneficial bacteria in the gut microbiome of low birth weight infants [161]. In a study by Radke et al., infants receiving formula supplemented with both prebiotics (bovine milk-derived oligosaccharides) and probiotics (*Bifidobacterium animalis* sp. lactis) have higher levels of *Bifidobacterium* and *Lactobacilli*, lower faecal pH, and higher faecal secretory IgA compared to infants receiving unsupplemented formula. The Committee on Nutrition of the European Society for Paediatric Gastroenterology and Nutrition (ESPGHAN) and the ESPGHAN Working Group for Probiotics and Prebiotics recommends the use of *L. rhamnosus GG* (LGG) ATCC 53103 (at a daily dose ranging from 1 × 10^9^ CFU to 6 × 10^9^ CFU) and the combination of *B. infantis* Bb-02, *B. lactis* Bb-12, and *Str. thermophilus* TH-4 (at a daily dose of 3.0 to 3.5 × 10^8^ CFU of each strain) to reduce necrotizing enterocolitis (NEC) stage 2 or 3 in preterm infants [162]. These suggestions that adding prebiotics and probiotics to infant formula may bring the gut microbiome closer to that of breastfed infants and positively influence the immune system [163]. As the postbiotic effect dependents on the bacterial strain, thus, the safety, suitability, and specificity of the bacteria strain in formula milk should be further studied.

### 4.3. Differences in the Gut Microbial Composition between Breastfed and Formula-Fed Infants

The gut microbiome of infants who receive human milk differs from that of infants fed formula milk. Formula feeding in term infants has increased microbial diversity [164,165,166]. Studies have reported higher levels of *Bifidobacteria*—an essential inhabitant of a healthy microbiota, in breastfed infants compared to formula-fed infants [166,167,168,169]. Consequently, breastfed and formula-fed infants have different levels of faecal SCFAs, the main metabolites of HMOs fermentation, with a higher level in breastfed infants [170]. However, Wang et al. reported that although the overall microbiota in breastfed and formula-fed infants are different, the levels of *Bifidobacteria* in both breastfeeding and formula-feeding groups are similar, suggesting that some formula milk is capable of supporting the growth of *Bifidobacteria* [171]. In an effort to create a gut microbiota profile that is comparable to that in breastfed infants, advances have been made in the formulation and manufacturing of infant formula milk, such as optimizing the whey-to-casein ratio and supplementing the formula milk with prebiotics, which has been proven to increase the abundance of *Bifidobacterium* in formula-fed babies [146,172].

## 5. Impact of the Early-Life Gut Microbiome on Health and Diseases

### 5.1. Necrotising Enterocolitis

Necrotising enterocolitis (NEC) is a serious inflammatory disease of the gastrointestinal tract predominantly affecting premature infants [173]. The earlier the gestational age, the higher the mortality due to NEC [174]. Dysbiosis and delayed gut microbiota maturation in preterm infants are associated with a higher risk of necrotising enterocolitis [175,176,177]. Prematurity is an independent factor associated with an altered gut microbiome [178]. Moreover, preterm infants are often treated with antibiotics and prolonged hospital care, which may directly influence the gut microbiota [179]. Cong et al. investigated the stool samples of 29 stable/healthy preterm infants at the NICU. The study found that the *Proteobacteria* was the most abundant phylum. There was an increasing pattern of *Clostridium* and *Bacteroides*, and decreasing *Staphylococcus* and *Haemophilus* observed over time during early life (Table 2) [180].

A similar bacteria phylum was observed in a study by Arboleya and colleagues. The study compared faecal samples from full-term vaginal delivered breast-fed infants (FTVDBF) and VLBW preterm infants. The preterm infants had higher levels of *Firmicutes* and *Proteobacteria*, and decreased levels of *Bacteroidetes* and *Actinobacteria* when compared with FTVDBF infants. The concentration of faecal SCFAs is also lower in premature infants, suggesting an alteration in the functionality of the preterm infant gut microbiome besides the composition [181]. McMurtry et al. reported that a reduced gut microbial diversity and the absence or significantly low abundance of certain classes of bacteria, for instance, *Clostridia*, are associated with an increased risk and severity of NEC. This suggests that a diverse microbiota and specific taxa may reduce the inflammatory response leading to the disease [182].

Human milk appears to protect against NEC as human milk (maternal or donor) is associated with decreased incidence of NEC compared to the preterm formula [183,197,198,199]. Kimak et al. recommended a longer term of exclusive human milk intake to decrease the risk of developing necrotising enterocolitis as the occurrence of NEC was higher in premature infants who received human milk for less than a week compared to those who exclusively received breast milk for more than a week [200]. Pourcyrous et al. found significant levels of total faecal SCFAs in preterm babies who received only expressed human milk compared to those who received preterm formula and suggested that the increased concentration of SCFAs in infants receiving human milk is associated with reduced risk of NEC [184]. Gopalakrishna et al. revealed that IgA from human milk plays a vital role in developing infant gut microbiota in preterm infants and preventing NEC [201]. Specific HMOs in human milk may also contribute to the protection against NEC, as 2′-fucosyllactose and disialyllacto-N-tetraose have been shown to lessen the severity of the disease and improve survival in animal models [202,203]. In addition, a study by Autran et al. found a significantly lower concentration of disialyllacto-N-tetraose in human milk samples received by VLBW infants who developed NEC. The concentration of this particular HMO could predict the risk of NEC occurrence [185].

Probiotics have been used to prevent necrotising enterocolitis in very low birth weight preterm babies; however, there are contradicting results concerning their effectiveness, which might be attributable to the modes of infant feeding and probiotic strains. For example, Braga et al. and Chowdhury et al. reported that human milk supplemented with probiotics (*Bifidobacterium* and *Lactobacillus*) decreased the incidence of necrotising enterocolitis [204,205]. A recent study by Robertson et al. also showed that probiotics supplementation with *Bifidobacterium bifidum* and *Lactobacillus acidophilus* is associated with a reduction in risk of NEC and late-onset sepsis, irrespective of mode of feeding [186]. In contrast, Demirel et al. found probiotic supplementation (*Saccharomyces boulardii*) in human or formula milk ineffective in decreasing NEC incidence or death [187]. Saengtawesin et al. found no difference in the incidence of NEC between infants receiving human milk or formula milk supplemented with *Bifidobacterium bifidum* and *Lactobacillus acidophilus* and the group that did not receive probiotics supplementation [188]. Dang et al. compared the outcomes of preterm infants before and after commencing probiotics supplementation consisting of *Lactobacillus rhamnosus* and *Bifidobacterium infantis*. It was reported that probiotics resulted in better feeding tolerance and reduced extra-uterine growth restriction; however, there was no significant difference in the occurrence of NEC [206]. The European Society for Paediatric Gastroenterology Hepatology and Nutrition (ESPGHAN) Committee on Nutrition and the Working Group for Probiotics and Prebiotics reported that only a small number of the investigated probiotic strains or combinations of strains were found to be efficacious in decreasing morbidity and mortality. Although there is low certainty in evidence, the ESPGHAN position paper conditionally recommended using *Lactobacillus rhamnosus* GG ATCC53103 or the combination of *Bifidobacterium infantis* Bb-02, *Bifidobacterium lactis* Bb-12, and *Streptococcus thermophilus* TH-4 in lowering the risk of necrotising enterocolitis [207].

### 5.2. Obesity

The increasing prevalence of infant and childhood obesity is of concern, as obesity can harm health and quality of life. Moreover, children who are overweight or obese are more prone to becoming obese adults and developing chronic diseases [208]. It has been found that infant gut microbiota may predict the risk of excessive weight gain in childhood [190]. A large study by Scheepers et al. reported that early infancy gut microbial composition, particularly the abundance of *Bacteroides fragilis*, is significantly correlated with weight gain in children [189]. In addition, the levels of *Streptococcus* in infants have been found to positively correlate with childhood body mass index (BMI), whereas the levels of *Bifidobacterium* are negatively correlated with the later BMI [190,209,210]. Factors influencing the gut microbiome development in infancy, including mode of delivery and perinatal antibiotic exposure, have associations with childhood adiposity [211,212]. Caesarean section and use of antibiotics during the first six months of age are associated with higher body mass in later years as reported by Blustein et al. and Trasande et al., respectively [213,214]. However, Li et al. suggested that rather than antibiotic exposure, untreated infection—which can also disrupt the gut microbiome—during infancy is correlated with excessive weight gain in childhood [215].

Many studies have demonstrated that breastfeeding has a protective effect on childhood obesity, while some have provided contradictory results. Several studies have reported that breastfed children for at least six months have a lower risk of overweight or obesity in childhood compared to those who have never received breast milk [216,217,218]. Some studies revealed a dose-response relationship between the duration of breastfeeding during infancy and child overweight or obesity risk [219,220,221]. In contrast, some studies found no association and dose-response effect between breastfeeding and obesity in later years [222,223]. On the other hand, studies on formula-fed infants demonstrated that different types of infant formula might also affect the pattern of weight gain due to the energy balance mechanisms. A study by Weber et al. revealed that compared to infants who received formula milk with higher protein content, those who received formula with lower protein content had lower BMI and lowered risk of obesity at older age [224]. Early-life consumption of higher protein content has been associated with more rapid weight gain, resulting in higher adiposity [225]. In addition, a more significant proportion of infants fed with cow’s milk formula were fast weight gainers compared to infants receiving extensively hydrolysed formula [226]. There is an association between rapid weight gain during infancy and high BMI later in life [227].

### 5.3. Atopy

Atopy is characterized by a tendency to develop hypersensitivity reactions in which there is elevated immunoglobulin E (IgE) production in response to antigens or allergens. Some common atopic conditions include allergic rhinitis, asthma, atopic dermatitis, and food allergy [228]. The development of atopic disorders may be influenced by early-life gut microbiota. Studies have revealed that reduced gut microbial diversity in early infancy is associated with an increased risk of developing atopic diseases [229,230,231]. Penders et al. showed that *Clostridia* colonization in 5 and 13-week-old infants was associated with a higher risk of developing atopic dermatitis [191]. West et al. demonstrated that infants with IgE-associated atopic dermatitis have lower Gram-positive *Ruminococcaceae*, which is associated with excessive TLR2 response, suggesting that the infant gut microbial composition may be correlated with susceptibility to eczema through immune signalling modulation [192]. Ta et al. revealed that infants with atopic dermatitis have an altered developmental trajectory in their gut microbiome. There was an enrichment of *Enterobacteriaceae* at three weeks of life and a delay in *Bacteroidaceae* colonization, leading to an increase in *Enterobacteriaceae/Bacteroidaceae* ratio in infants with atopic dermatitis [232]. Arrieta et al. demonstrated a transient dysbiosis of the intestinal microbiota in children at risk of asthma during their first few months. These children have a lower abundance of *Lachnospira*, *Veillonella*, *Rothia*, and *Faecalibacterium* in their gut microbiome at three months old. Furthermore, inoculation of germ-free mice with these four microbes have been shown to reduce airway inflammation, suggesting that they may play a protective role in asthma development [233].

Studies have shown that human milk confers protection against the development of atopic diseases. A study involving 3296 children from the Canadian Healthy Infant Longitudinal Development birth cohort reported that formula feeding and mixed feeding are associated with a higher risk of asthma by three years of age compared to exclusive breastfeeding during the first three months of life [234]. While the risk of developing childhood asthma was higher in infants delivered by caesarean section without medical indication, exclusive breastfeeding in the first six months of life may reduce this risk [235]. Elbert et al. found a slight increase in the risk of developing atopic dermatitis in children who were breastfed for a shorter duration and those who were nonexclusively breastfed [193]. A recent clinical report from the American Academy of Paediatrics concluded that exclusively breastfed infants during the first three to four months of life had a reduced risk of atopic dermatitis up to two years of age. In addition, breastfeeding for a more extended period, regardless of exclusivity, confers protection against asthma even beyond age five. There was no evidence that exclusive breastfeeding for more than three to four months offers any benefits in preventing atopic disorders [236]. There is conflicting evidence on the preventive effect of partially hydrolysed whey formula, as supplement or substitute to human milk, on atopic disorders in high-risk children [237,238,239].

Evidence on the efficacy of prebiotic supplementation in atopic disorders prevention is inconsistent. Arslanoglu et al. revealed that extensively hydrolysed whey formula supplemented with short-chain galacto-oligosaccharides and long-chain fructo-oligosaccharides (9:1; 8 g/L) significantly decreased the risk of any atopic manifestations and atopic dermatitis up to five years of age [194]. Wopereis et al. demonstrated that partially hydrolysed formula supplemented with neutral short-chain galacto-oligosaccharides, long-chain fructo-oligosaccharides (9:1; 0.68 g/100mL) and pectin-derived acidic oligosaccharide (0.12 g/100mL) brought the gut microbiome of formula-fed infants closer to that of breast-fed infants [240]. However, Boyle et al. reported that adding a specific prebiotic mixture to partially hydrolysed whey formula was ineffective in preventing atopic dermatitis in high-risk children in the first year of life [241].

The use of probiotic supplementation to prevent infants’ allergies remains controversial. Rautava et al. evaluated the effect of maternal probiotic supplementation with *Lactobacillus rhamnosus* LPR + *Bifidobacterium longum* BL999 or *Lactobacillus paracasei* ST11 + *Bifidobacterium longum* BL999 during the last two months of pregnancy and the subsequent two months during breastfeeding. The study reported that the risk of infants developing atopic dermatitis in the first two years of life was significantly decreased [195]. Wickens et al. showed that *Lactobacillus rhamnosus* HN001 protected infants against atopic dermatitis if administered to mothers from 35 weeks to 6 months postpartum if breastfeeding and to the infants during their first two years of life [196]. Conversely, Allen et al. found that probiotics (*Lactobacillus salivarius* CUL61, *Lactobacillus paracasei* CUL08, *Bifidobacterium animalis* subspecies *lactis* CUL34, and *Bifidobacterium bifidum* CUL20) given to mothers from 36 weeks of pregnancy until delivery and to infants until 6 months of age did not prevent atopic dermatitis in the first two years of life [242]. Using different probiotic strains, Loo et al. and Cabana et al. evaluated the effect of infant probiotic supplementation during the first six months of life. They found that early probiotic supplementation did not prevent atopic diseases at two and five years of age [243,244]. In short, it is still uncertain whether prebiotics or probiotics supplementation helps prevent atopic diseases owing to the heterogeneity between studies, particularly concerning the types of prebiotics or probiotics used and the duration of supplementation.

## 6. Conclusions

**Figure 1:**
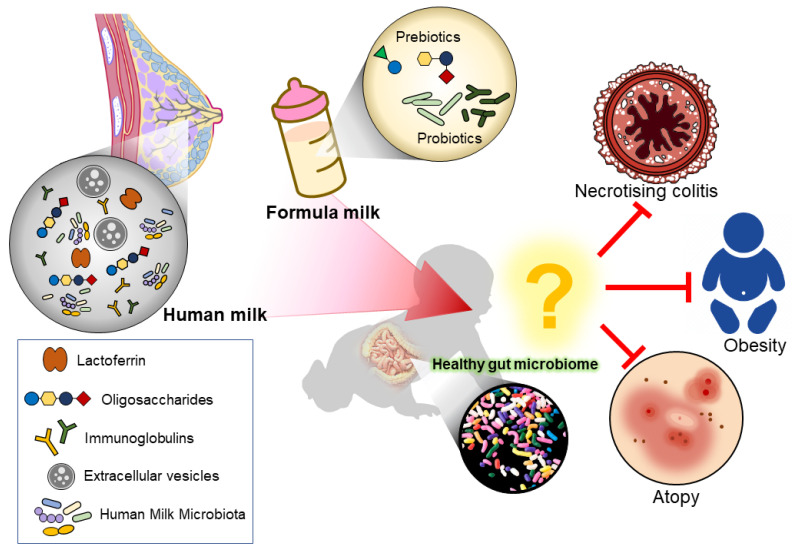
Illustration of how the different feeding mode modulates the infant gut microbiome. Human milk naturally contains lactoferrin, oligosaccharides, immunoglobulins, extracellular vesicles, and human milk microbiota, which aids in modulating a healthy infant gut. While formula milk often has additional supplements added to mimic human milk.

The infant gut microbiota is complex and can be modulated by many endogenous and exogenous factors (Figure 1). The feeding mode during infancy has been demonstrated to be one of the most important factors in determining the development and establishment of gut microbial composition. Human milk is an excellent source of infant nutrition and has been shown to provide many health benefits; thus, breastfeeding should be encouraged. However, in some circumstances, mothers may not be able to breastfeed or decide to formula feed their babies. Therefore, continuous advances and innovations in infant formula development are important to produce the best alternative for infants who are not breastfed.

Studies have suggested that supplementing prebiotics or probiotics in infant formula brings the gut microbiome of formula-fed infants closer to that of breastfed infants. However, its clinical efficacy in preventing diseases, such as necrotising enterocolitis and atopic disorders, should be further explored and studied. The contradictory results concerning their effectiveness might be attributable to different oligosaccharides or probiotic strains in various studies. Therefore, more evidence is needed to establish its clinical efficacy and determine the best prebiotic or probiotic strains, their doses, and duration of supplementation.

## Figures and Tables

**Table 1:** The human milk constituents and concentrations [27,54,55].

Water	87–88%	
**Macronutrients**		
Protein	1% (8–10 g/L)	
Carbohydrates	7% (60–70 g/L)	
Fat	3.8% (35–40 g/L)	
**Micronutrients**		
Fat-Soluble Vitamins		
Vitamin A	0.3–0.6 mg/L	
Vitamin D	40 IU/L	
Vitamin E	3–8 mg/L	
Vitamin K	2–3 μg/L	
Water-Soluble Vitamins		
Ascorbic acid	100 mg/L	
Vitamin B1	200 μg/L	
Vitamin B2	0.35–0.39 mg/L	
Niacin	1.8–6 mg/L	
Vitamin B6	0.09–0.31 mg/L	
Vitamin B12	0.5–1 μg/L	
Folate	80–140 μg/L	
Minerals		
	Colostrum	Mature Milk
Calcium	250 mg/L	200–250 mg/L
Magnesium	30–35 mg/L	30–35 mg/L
Phosphorus	120–160 mg/L	120–140 mg/L
Sodium	300–400 mg/L	150–250 mg/L
Potassium	600–700 mg/L	400–550 mg/L
Iron	05–1.0 mg/L	0.3–0.7 mg/L
Chloride	600–800 mg/L	400–450 mg/L
Zinc	5–12 μg/L	1–3 μg/L

**Table 2:** Summary of early-life gut microbiome on health and diseases.

Subjects	Baseline Gut Microbiome Composition Compared to Control Group	Intervention	Key Findings of the Study	Reference
**Necrotising entercolitis (NEC)**				
Preterm infants	Proteobacteria most abundant phylum	N.A.	There was a significant change observed in the gut microflora. Increased *Proteobacteria*, *Clostridium*, and *Bacteroides*, and decreasing *Staphylococcus* and *Haemophilus* were observed over time	[180]
Full-term vaginal delivered breast-fed infants (FTVDBF) and VLBW preterm infants	Higher levels of *Firmicutes* and *Proteobacteria*, and decreased levels of *Bacteroidetes* and *Actinobacteria*	N.A.	There was a significant change observed between the full-term vaginal delivered breast-fed infants (FTVDBF) and VLBW preterm infants	[181]
Preterm infants	Lower level of *Clostridia*	N.A.	There was a significant change observed and this is associated with an increased risk and severity of NEC	[182]
Infants diagnosed with NEC withing first 30 days of life	N.A.	Human milk	Infants who received human milk for >7 days had decreased risk of NEC compared to infants who received human milk for less than 1 week	[183]
Preterm infants	N.A.	Human milk	Total SCFA concentrations were higher for human milk-fed infants than those for preterm-formula milk-fed infants. This is associated with reduced risk of NEC	[184]
		Preterm-formula milk		
Very low birthweight infants (VLBW)	N.A.	Human milk	A lower concentration of disialyllacto-N-tetraose in human milk samples received by VLBW infants who developed NEC. Eight infants in the cohort developed NEC (Bell stage 2 or 3)	[185]
Very low birthweight infants (VLBW)	N.A.	Human milk and formula milk (for full feeds) supplemented with probiotic	*Bifidobacterium bifidum* and *Lactobacillus acidophilus* is associated with a reduction in the risk of NEC, late-onset sepsis, and mortality irrespective feeding mode	[186]
Preterm babies <32 weeks and VLBW	N.A.	Human milk or formula milk supplemented with *S.boulardii*	*Saccharomyces boulardii* supplementation at a dose of 250 mg/day was not effective at reducing the incidence of death or NEC in VLBW infants, it improved feeding tolerance and reduced the risk of clinical sepsis	[187]
Preterm infants	N.A.	Human milk or formula milk supplemented with probiotic	No difference in the incidence of NEC between infants receiving human milk or formula milk supplemented with *Bifidobacterium bifidum* and *Lactobacillus acidophilus* and the group that did not receive probiotics supplementation	[188]
**Obesity**				
Termed infants	*Bacteroides fragilis*	N.A.	Colonization with *B. fragilis* group was borderline significantly associated with a higher BMI	[189]
Infants	*Streptococcus*	N.A.	Colonization of *Streptococcus* was significantly higher in the first months of life and has been associated with higher adiposity and BMI	[190]
**Atopy**				
Newborns with a single or double heredity for atopy	Clostridia	N.A.	Clostridium colonization in neonates is associated with an increased risk of atopic dermatitis	[191]
10 children with IgE-associated eczema and 10 nonallergic children	*Ruminococcaceae*	N.A.	Relative abundance of Gram-positive *Ruminococcaceae* was lower at one week of age in infants developing IgE-associated eczema, compared with controls	[192]
2–6 months infants who are breastfed	N.A.	Human milk	Shorter breastfeeding duration was associated with an overall increased risk of eczema	[193]
Healthy term infants at risk of atopy	N.A.	Formula milk	Hydrolysed whey formula supplemented with short-chain galacto-oligosaccharides and long-chain fructo-oligosaccharides (9:1; 8 g/L) significantly decreased the risk of atopic	[194]
Mother–infant pairs	N.A.	*Lactobacillus rhamnosus* LPR + *Bifidobacterium longum* BL999 or *Lactobacillus paracasei* ST11 + *Bifidobacterium longum* BL999 for the maternal until delivery. Then babies are breast-fed	The risk of developing eczema during the first 24 months of life was significantly reduced in infants of mothers receiving LPR + BL999 (odds ratio [OR], 0.17; 95% CI, 0.08–0.35; *p* < 0.001) and ST11+BL999	[195]
Infants	N.A.	*Lactobacillus rhamnosus* HN001	Maternal supplementation from 35 weeks gestation until 6 months of breastfeeding and infant supplementation until two years with *Lactobacillus rhamnosus* HN001 reduced the prevalence of eczema	[196]

N.A.: Not applicable.

## Data Availability

Not applicable.
